# Pulmonary embolism in a post-COVID-19 patient: a critical diagnostic challenge

**DOI:** 10.25122/jml-2025-0101

**Published:** 2025-09

**Authors:** Diana-Alexandra Mîțu, Daian-Ionel Popa, Florina Buleu, Carmen Williams, Dumitru Sutoi, Daniel-Florin Lighezan, Ovidiu Alexandru Mederle

**Affiliations:** 1 Department of Internal Medicine I, Victor Babeș University of Medicine and Pharmacy, Timisoara, Romania; 2 Doctoral School, Faculty of General Medicine, Victor Babeș University of Medicine and Pharmacy Timisoara, Timisoara, Romania; 3 Department of Cardiology, Victor Babeș University of Medicine and Pharmacy, Timisoara, Romania; 4 Emergency Municipal Clinical Hospital, Timisoara, Romania; 5 Department of Surgery, Emergency Discipline, Victor Babeș University of Medicine and Pharmacy, Timisoara, Romania

**Keywords:** COVID-19, pulmonary thromboembolism, SARS-COV2

## Abstract

The most severe clinically recognized complication of venous thromboembolism (VTE), pulmonary embolism (PE), can be difficult to diagnose due to its nonspecific symptoms. The overlapping clinical symptoms of severe acute respiratory syndrome coronavirus 2 (SARS-CoV-2) infection that causes Coronavirus 2019 (COVID-19) and PE can make it difficult to differentiate between one and the other. Therefore, PE diagnosis can be delayed or missed in patients with COVID-19, resulting in critical consequences for patient safety and outcomes. A 70-year-old woman presented to our Emergency Department with dyspnoea and chest pain. On admission, she had peripheral O2 saturation (SpO2) of 94% on 6 l/min O2, pain, and an increase in the volume of the right lower limb. Anamnesis revealed that she had been discharged two weeks earlier from the Infectious Diseases Department, where she was admitted for SARS-CoV-2 infection. Venous Doppler ultrasound of the right limb revealed complete thrombosis in the common femoral, popliteal, and small saphenous veins. The computed tomography angiography of the pulmonary artery revealed defects suggestive of pulmonary thromboembolism, visualized in the pulmonary artery trunk, bilateral pulmonary arteries, and various lobes. In patients with a recent history of COVID-19, pulmonary thromboembolism must always be considered as a critical differential diagnosis. Timely recognition and intervention are vital, as they can significantly influence the patient's prognosis and overall outcome through prompt diagnosis and appropriate treatment.

## Introduction

The rapid spread of coronavirus cases led the World Health Organization (WHO) Director-General to declare a pandemic on March 11, 2020. The disease, officially known as coronavirus disease 2019 (COVID-19), is caused by the severe acute respiratory syndrome coronavirus 2 (SARS-CoV-2). Although initially thought to affect the lungs primarily, it has since become evident that COVID-19 can involve multiple organ systems [1]. The disease poses a risk for thrombotic complications in the venous and arterial circulation due to inflammation, platelet activation, endothelial dysfunction, and stasis [2]. A study published in the Journal of the American College of Cardiology analyzed 328 patients and found that 22% developed pulmonary embolism (PE) within one month of SARS-CoV-2 infection [3].

Venous thromboembolism (VTE), which includes deep vein thrombosis (DVT) and pulmonary embolism, represents a significant contributor to morbidity and mortality on a global scale. Among the complications associated with VTE, pulmonary embolism is the most critical. It poses a considerable risk to life, frequently manifesting with nonspecific symptoms such as chest pain, dyspnoea, and hypoxia [4]. The clinical manifestations of pulmonary embolism bear considerable resemblance to those of COVID-19, which is caused by SARS-CoV-2, thereby complicating the process of achieving a timely and accurate diagnosis [5].

COVID-19 is linked to a hypercoagulable condition that markedly elevates the likelihood of thromboembolic incidents, even among patients who do not exhibit critical illness [6]. Within this framework, pulmonary embolism (PE) may be overlooked or mistakenly attributed to complications related to COVID-19, resulting in delays in suitable treatment and negative outcomes [5].

This case report details a patient who recently experienced a SARS-CoV-2 infection and initially exhibited symptoms indicative of potential post-COVID pulmonary complications. However, the patient was later diagnosed with significant pulmonary embolism. This case emphasizes the necessity of sustaining a heightened clinical awareness for pulmonary embolism in post-COVID individuals presenting with respiratory or limb-related symptoms and highlights the urgency for timely diagnostic imaging and intervention.

## Case presentation

Patient R.D., a 70-year-old woman weighing 80 kg, had been diagnosed with SARS-CoV-2 infection two weeks earlier and was hospitalized in the Infectious Diseases Clinic. However, she requested early discharge and did not follow the prescribed treatment at home. After a short period, she was admitted to the Emergency Department (ED) of Municipal Clinical Hospital, Timisoara, Romania, with acute respiratory failure.

Physical examination revealed altered general condition, hyperaemic skin, and edema in the right thigh and leg. Pulses were absent in the posterior tibial and dorsalis pedis arteries. Vital signs included blood pressure 125/95 mmHg, heart rate 96 beats/min, temperature 36.5 °C, and spontaneous oxygen saturation (SaO_2_) of 94%. The Glasgow Coma Score (GCS) was 15/15, and no added pulmonary rales were detected. The patient complained of dyspnoea, chest pain, and an increase in the volume of the right lower limb; symptoms started two days before presentation in the ED with progressive worsening. Based on the reported history, clinical examination, and elevated D-dimer levels, pulmonary thromboembolism was suspected. A computed tomography angiography of the pulmonary arteries and a venous Doppler ultrasound were performed to rule out the presence of thrombi in the deep venous system.

**Figure 1:**
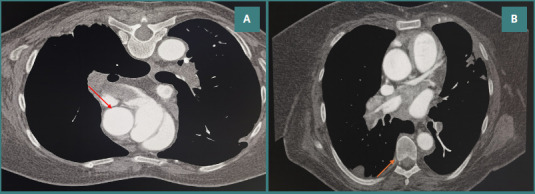
Chest computed tomography pulmonary angiography. A, Axial CTPA image showing a large intraluminal filling defect (red arrow) within the right pulmonary artery, consistent with acute pulmonary embolism. B, Axial CTPA image demonstrating a segmental pulmonary embolism (orange arrow) in the left lower lobe pulmonary artery branch.

**Figure 2:**
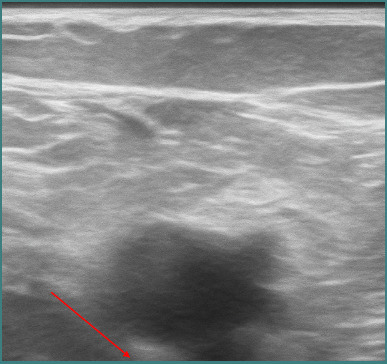
Venous Doppler ultrasound

The chest CT scan revealed filling defects suggestive of pulmonary thromboembolism visualized in the trunk of the pulmonary arteries, as well as in the bilateral pulmonary arteries and in the right lower lobe (RLL), right middle lobe (RML), left upper lobe (LUL), and left lower lobe (LLL), including their respective segments. The upper lobes were less prominently affected (Figure 1AB). Venous Doppler ultrasound revealed complete thrombosis in the common femoral, popliteal, and small saphenous veins (Figure 2).

The patient was hospitalized in the Department of Internal Medicine for specialized treatment and additional investigations, during which cardiac and abdominal ultrasounds were performed.

**Figure 3:**
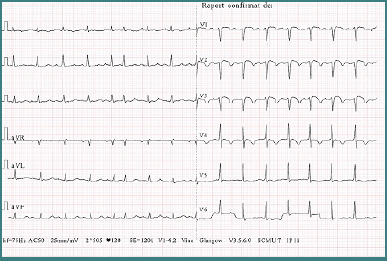
The EKG of the patient

The electrocardiogram (EKG) showed a sinus rhythm with an intermediate QRS axis, a heart rate of 120 beats/min, T-wave inversions in leads I and V1–V5, and a premature atrial complex (Figure 3).

Laboratory investigations revealed markedly elevated inflammatory markers, including C-reactive protein (CRP) 202.4 mg/L (normal <5 mg/L), erythrocyte sedimentation rate (ESR) 115 mm/h (normal <20 mm/h), and D-dimer 5100 ng/mL FEU (normal <500 ng/mL FEU). Anti–SARS-CoV-2 antibody testing was positive, with IgG 9.58, IgM 10.5, and IgA 8.83 (reference: negative <1, positive >1 for all antibody classes).

On admission, anticoagulation was initiated with intravenous fondaparinux, 7.5 mg daily for 7 days. This was followed by a transition to oral apixaban, 5 mg twice daily. Supportive therapy included anti-inflammatory agents, statins, and electrolyte replacement solutions.

The clinical progression was positive, with gradual improvement in respiratory and vascular symptoms. Following a nine-day hospitalization in the Internal Medicine Department, the patient was discharged in stable condition.

At discharge, it was advised that oral anticoagulant therapy be maintained. The follow-up protocol encompassed a repeat venous Doppler ultrasound of the lower extremities one month following discharge, as well as a chest angio-computed tomography scan every six months. Additionally, regular assessments were arranged within the Internal Medicine Department to evaluate disease progression, therapeutic response, and the emergence of potential complications.

## Discussion

COVID-19 is increasingly recognized as a prothrombotic condition that predisposes patients to thromboembolic complications in both the venous and arterial systems. The underlying pathophysiology is thought to involve a combination of endothelial dysfunction, cytokine storm, hypoxia-induced thrombosis, and the direct impact of the virus on the coagulation cascade. Together, these elements lead to a state of hypercoagulability, which can persist even after the acute phase of infection [7].

Numerous studies have recorded a heightened occurrence of venous thromboembolism among hospitalized patients with COVID-19, especially in those necessitating intensive care [8-10]. Nevertheless, thrombotic complications may also arise in patients who are not critically ill and can manifest after discharge [11], as illustrated by this case. This underscores the need for clinical vigilance during the post-acute phase of COVID-19, particularly among elderly individuals and those with additional risk factors for thrombosis.

The literature also identifies general mechanisms such as inflammation, endothelial dysfunction, hypoxia, and stasis as contributors to thromboembolic events in COVID-19 [12,13]. However, a more detailed and individualized examination uncovers additional significant factors. In this instance, markedly elevated inflammatory markers (CRP: 202.4 mg/L, ESR: 115 mm/h) indicated a vigorous cytokine-mediated response, which likely plays a crucial role in initiating immunothrombosis—a phenomenon driven by the interplay of inflammatory cells, platelets, and the coagulation cascade. The notably high D-dimer level (5100 ng/mL) suggests extensive fibrin formation and degradation, aligning with COVID-19-associated coagulopathy (CAC). Furthermore, the patient's seropositivity for anti-SARS-CoV-2 IgG, IgM, and IgA antibodies indicates an ongoing immune response that may have contributed to endothelial activation and a persistent prothrombotic condition. It is essential to emphasize that the absence of thromboprophylaxis following initial discharge, coupled with the presence of extensive lower limb thrombosis, underscores the necessity for tailored post-COVID risk evaluations and anticoagulation strategies. These observations support the hypothesis that, in addition to general mechanisms, the unique immune and coagulation responses of individual patients play a pivotal role in post-infectious thromboembolic complications.

This case highlights the difficulties that clinicians encounter when assessing post-COVID-19 patients exhibiting nonspecific symptoms, including dyspnea and chest pain. The significant overlap of these symptoms with typical presentations of pulmonary embolism may result in delays in diagnosis [14]. In the instance of our patient, a recent history of SARS-CoV-2 infection, combined with clinical indications suggestive of deep vein thrombosis, necessitated further imaging investigations, which ultimately validated the presence of extensive venous thrombosis and bilateral pulmonary embolism.

Distinguishing post-COVID dyspnea from pulmonary embolism presents a significant diagnostic challenge due to the substantial overlap in clinical symptoms [14,15]. Additional research indicates that the post-COVID-19 condition, particularly pulmonary embolism, disproportionately impacts women and those with preexisting conditions such as cancer or diabetes. The symptom overlap between post-COVID-19 condition and pulmonary embolism complicates the diagnostic process, underscoring the importance of diagnostic tools such as lung perfusion scans. Although many laboratory findings may return to normal over time, the risk of pulmonary embolism can remain, necessitating continued vigilance. The lower quality of life scores, especially in the areas of pain/discomfort and anxiety/depression, underscore the necessity for comprehensive, long-term care that includes mental health support [15].

Persistent shortness of breath, chest discomfort, and fatigue are common in post-acute COVID-19 syndrome, but they may also signal a delayed thromboembolic complication. In this context, the presence of certain clinical indicators should raise suspicion for PE, including unilateral limb swelling, pleuritic chest pain, unexplained tachycardia, or disproportionate oxygen desaturation on minimal exertion. In our female patient, the acute onset of dyspnea combined with right lower limb edema and elevated D-dimer levels warranted immediate imaging, which confirmed the diagnosis. To support earlier recognition of PE in similar patients, clinicians should employ risk stratification tools, such as the modified Wells or Geneva scores, alongside targeted laboratory testing (e.g., D-dimer), and provide prompt access to CT pulmonary angiography when indicated. Incorporating a standardized protocol for evaluating dyspnea in post-COVID patients, particularly those recently hospitalized or non-adherent to thromboprophylaxis, may help reduce diagnostic delays and improve clinical outcomes.

In accordance with established guidelines, anticoagulation therapy was initiated at admission with intravenous fondaparinux, 7.5 mg daily, and continued for 7 days. Fondaparinux, recognized as a selective factor Xa inhibitor, is frequently employed in acute scenarios due to its reliable pharmacokinetic properties, minimal risk of heparin-induced thrombocytopenia (HIT), and the convenience of once-daily administration. This medication proves particularly advantageous for patients presenting with a significant thrombotic burden or where parenteral anticoagulation is preferred in the early stages of treatment. After achieving initial stabilization, the patient was switched to apixaban, an oral direct factor Xa inhibitor, prescribed at a dosage of 5 mg twice daily. This transition follows a step-down approach that is consistent with international protocols, including those issued by the American College of Chest Physicians (CHEST) and the European Society of Cardiology (ESC), which advocate for the use of direct oral anticoagulants (DOACs) such as apixaban for the long-term outpatient management of venous thromboembolism, given their effectiveness, favorable safety profile, and the simplicity of administration without necessitating routine laboratory monitoring [16,17].

Alongside anticoagulation therapy, the patient was administered additional supportive treatments, including anti-inflammatory medications, statins, and solutions for electrolyte rebalancing. These interventions were designed to manage inflammation, enhance cardiovascular health, and rectify metabolic imbalances frequently seen following COVID-19 [18]. The clinical progress was positive, demonstrating a gradual improvement in respiratory and vascular symptoms. After a nine-day stay in the Internal Medicine Department, the patient was discharged in stable condition.

Oral anticoagulation with apixaban was maintained for the care following discharge, and a comprehensive follow-up plan was formulated. This plan encompassed: (1) a repeat venous Doppler ultrasound of the lower extremities one month after discharge to evaluate thrombus resolution or progression; (2) chest angio-computed tomography scans conducted every 6 months to detect potential recurrence or chronic thromboembolic complications; and (3) consistent outpatient follow-up at the internal medicine clinic for ongoing assessment and medication management. This follow-up protocol aligns with guidelines for patients susceptible to recurrent thromboembolic events, particularly in light of recent COVID-19, during which long-term vascular complications have been increasingly documented [19].

In light of the sequelae of the global burden posed by COVID-19, clinicians should integrate post-discharge thromboprophylaxis protocols for individuals identified as being at high risk. Research has indicated that the risk of thromboembolism may extend beyond the acute phase of the infection, particularly affecting older adults and individuals with comorbid conditions such as obesity, cancer, or a history of venous thromboembolism [20,21]. This consideration becomes increasingly pertinent within the framework of long COVID, a condition marked by lingering symptoms that may persist for weeks or months following the initial SARS-CoV-2 infection. New evidence points to the possibility that the hypercoagulable state linked to COVID-19 could not only lead to acute thromboembolic incidents but also result in sustained endothelial dysfunction and microvascular complications associated with long COVID [21,22].

The significance of comprehensive risk assessment in patients recovering from COVID-19 is underscored by our case, particularly in instances where new or unexplained cardiopulmonary symptoms are present. The diagnostic challenge is exacerbated by the symptom overlap between long COVID, characterized by fatigue, dyspnea, and chest discomfort, and pulmonary embolism, which raises the likelihood of missed or postponed treatment. This situation underscores the necessity of sustaining a heightened level of suspicion for pulmonary embolism within the differential diagnosis of post-COVID conditions, particularly when there are accompanying indications of deep vein thrombosis or disproportionate oxygen desaturation.

## Conclusion

This case underscores the imperative to regard pulmonary embolism as a potential differential diagnosis in individuals with a recent COVID-19 history, especially when they exhibit respiratory symptoms or signs indicative of deep vein thrombosis. The diagnostic process can be complicated, and treatment may be delayed due to the overlapping clinical manifestations of COVID-19, long COVID, and pulmonary embolism, which could result in serious consequences. It is essential for clinicians to maintain a heightened level of suspicion and to promptly assess any unexplained cardiopulmonary symptoms in patients recovering from COVID-19. The early identification and prompt initiation of suitable anticoagulation therapy are crucial for improving patient outcomes. Additionally, a personalized risk evaluation for post-discharge thromboprophylaxis should be considered for high-risk patients as part of a thorough approach to care during the recovery period from COVID-19.
